# Development of *Neonectria punicea* Pathogenic Symptoms in Juvenile *Fraxinus excelsior* Trees

**DOI:** 10.3389/fpls.2020.592260

**Published:** 2020-12-23

**Authors:** Dragan Karadžić, Zoran Stanivuković, Slobodan Milanović, Katarzyna Sikora, Zlatan Radulović, Vladimír Račko, Monika Kardošová, Jaroslav Ďurkovič, Ivan Milenković

**Affiliations:** ^1^The Chair of Forest Protection, Department of Forestry, University of Belgrade-Faculty of Forestry, Belgrade, Serbia; ^2^Department of Integrated Protection of Forest Ecosystems, Forestry Faculty, University in Banja Luka, Banja Luka, Bosnia and Herzegovina; ^3^Department of Forest Protection and Wildlife Management, Faculty of Forestry and Wood Technology, Mendel University in Brno, Brno, Czechia; ^4^Department of Forest Protection, Forest Research Institute-IBL, Raszyn, Poland; ^5^Department of Forest Protection, Institute of Forestry Belgrade, Belgrade, Serbia; ^6^Department of Wood Science, Technical University in Zvolen, Zvolen, Slovakia; ^7^Department of Phytology, Technical University in Zvolen, Zvolen, Slovakia; ^8^Phytophthora Research Centre, Faculty of Forestry and Wood Technology, Mendel University in Brno, Brno, Czechia

**Keywords:** common ash, ash dieback, collar necrosis, pathogenicity, SEM, X-ray microCT imaging

## Abstract

When monitoring the state of health of *Fraxinus excelsior* trees, unusual symptoms were discovered within a *F. excelsior* plantation in Bosnia and Herzegovina. These symptoms included the appearance of necrosis and cankers in the basal parts of the trees, followed by the formation of fruiting bodies, however, none of these symptoms were found in the crowns. After sampling and isolation of the necrotic parts from the stem base, pathogen *Neonectria punicea* was isolated and identified from the characteristics of pure cultures, morphology of the fruiting bodies, and from multilocus sequencing. In field conditions, juvenile *F. excelsior* trees were inoculated with two *N*. *punicea* isolates obtained from the necrotic tissues of both juvenile *F. excelsior* and mature *Fagus sylvatica* trees. In both isolates, 12 months post inoculation, the lengths and widths of the necroses were significantly larger compared to the control. Necroses of significantly larger lengths, widths and surfaces were found again in both tested isolates 24 months post inoculation. In the case of the *F. excelsior* isolate, the lengths of the necroses at both the stem base and at breast height increased by 1.6 times, whereas the *F. sylvatica* isolate increased in size by up to 1.7 and 1.8 times, respectively. Trees inoculated without a previous bark wound showed no symptoms, similar to the control trees. Scanning electron microscopy and X-ray micro-computed tomography imaging revealed that *N. punicea* hyphae penetrated from the cankers to the woody outermost annual growth ring and that hyphae were present mostly in the large earlywood vessels and rarely in the axial parenchyma cells. Hyphae also spread radially through the pits in vessels. The infected trees responded with the formation of tyloses in the vessels to prevent a rapid fungal spread through the axial vascular transport pathway. The ability of *N. punicea* to cause necroses in juvenile ash trees was demonstrated for the first time during this study. It poses a serious threat to planted forests and natural regenerations of *F. excelsior* especially if *F. sylvatica* is considered as a possible inoculum reservoir for future infections. This pathogen should be integrated within future ash resistance or breeding programs.

## Introduction

Common ash (*Fraxinus excelsior* L.) can be found in almost all European countries, but its distribution varies considerably in different parts of Europe. In many areas, it is a keystone floodplain species; in the Balkan peninsula, this species most commonly occurs in submontane and montane forests, beyond rivers and stream valleys (e.g., in Serbia, it is typically found in *Acero-Fraxinetum serbicum* associations; Jovanović, 1971). In addition to being an important tree species for the forest industry, common ash is a favorite choice for parks, alleys, and gardens. Its two varieties (var. *glabra* and var. *pendula*) are used in the parks of the entire region and are commonly grown in numerous nurseries.

In the past 25 years, the exotic pathogenic fungus *Hymenoscyphus fraxineus* Baral, Queloz, and Hosoya has been the reported cause of common ash dieback across Europe (Kowalski, 2006; Gross et al., 2014; Keča et al., 2017; Milenković et al., 2017). Numerous studies have described the typical symptoms caused by this pathogenic fungus, including leaf-wilting, general necrosis, premature shedding of leaves, necrosis and lesions on the shoots and branches, increased crown transparency, and dieback (Kowalski, 2006; Bakys et al., 2009; Gross et al., 2014; Kräutler and Kirisits, 2014). Additionally, cankers and lesions at the stem bases of declining ash trees were reported, and these symptoms were initially assigned to different causal agents, mostly secondary pathogens such as *Armillaria* spp. (Lygis et al., 2005; Skovsgaard et al., 2010; Bakys et al., 2011; Enderle et al., 2013) or species from the *Phytophthora* genus (Orlikowski et al., 2011). However, Husson et al. (2012) demonstrated the presence of *H. fraxineus* in infected ash collar tissues for the first time and suggested that this pathogen could be the cause of these symptoms. These findings were confirmed in subsequent studies (Chandelier et al., 2016; Marçais et al., 2016; Enderle et al., 2017; Langer, 2017; Meyn et al., 2019).

Another pathogen, *Neonectria punicea* (J.C. Schmidt: Fr.) Castlebury and Rossman, closely related to the beech bark disease (BBD) phenomenon (Castlebury et al., 2006) was recorded in necrotic ash tissues (Langer, 2017; Meyn et al., 2019) in addition to *H. fraxineus*. This species is assumed to be endophytic and develops as a secondary pathogen in ash collar tissue after infection with ash dieback fungus (Meyn et al., 2019); however, the precise mechanisms of its aggressiveness and ability to cause cankers and lesions on ash collars remain unknown to date (Langer, 2017).

With respect to the health status of common ash in Bosnia and Herzegovina (B&H), *H. fraxineus* was first recorded in 2009 in young plants in one planted forest in the western part of the country (Stanivuković et al., 2014). It was also registered in a nursery in the central part of B&H (Treštić and Mujezinović, 2013) in 2013. After these occurrences, the disease quickly spread to almost all the natural stands of common ash in B&H (Stanivuković, unpublished). However, planted common ash forest showing declining symptoms different to those typically caused by ash dieback fungus was recorded in the western part of the country in 2014 during monitoring of ash dieback in B&H. Specifically, bark necrosis and cankers were exclusively recorded at the stem bases of trees, whereas no lesions were recorded on the shoots, branches, and upper parts of the stems. Moreover, following the appearance of bark cankers, numerous stromata were formed on the dead parts of the bark with masses of mostly red-colored ascomycetous perithecia fruiting bodies.

The objectives of this study were to (i) isolate and identify the cause of the symptoms observed in this plantation; (ii) test the pathogenicity of the isolated organism and compare its aggressiveness with similar isolates originating from necrotic European beech tissue; (iii) reveal whether the pathogen can infect the tree through uninjured bark, or whether the bark has to be previously damaged; and (iv) observe pathogen development and tree responses following infection.

## Materials and Methods

### Study Area and Disease Symptoms

**FIGURE 1:**
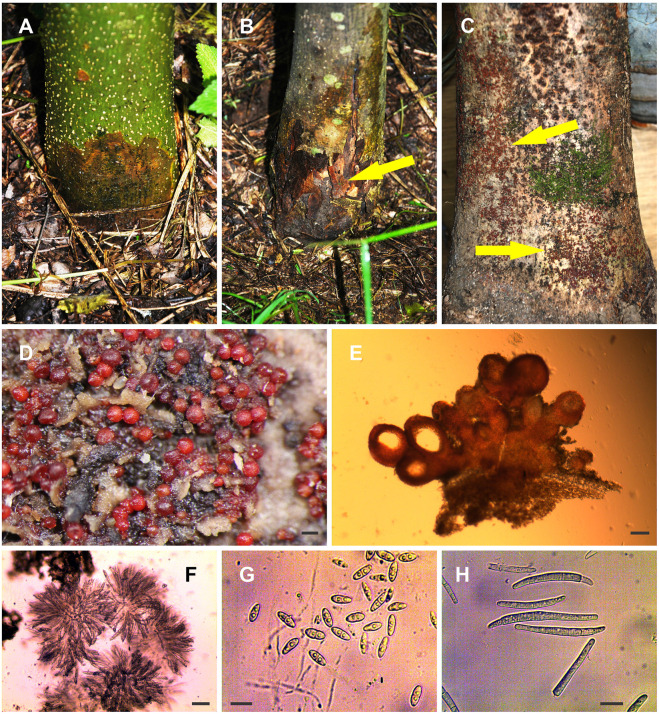
Recorded symptoms and fungal morphology. **(A,B)** Root collar necrosis (arrows indicate the positions of perithecial stromata). **(C,D)** The perithecia on the necrotic bark. **(E)** Cross section of perithecial stroma. **(F)** Asci with ascospores. **(G)** Ascospores. **(H)** Macroconidia (from culture). Scale bars: **(D)** = 200 μm, **(E)** = 100 μm, **(F)** = 50 μm, **(G,H)** = 10 μm.

The study was carried out in a plantation of common ash in the western part of B&H (44°41′11.87″N; 16°32′23.21″E). This plantation is located at an altitude of around 410–420 m a.s.l., and the site is exposed to relatively low temperatures (Supplementary Table S1) and satisfactory precipitation (Supplementary Table S2). The plantation was established on 0.5 ha, with over 2,000 seedlings planted at a spacing of 1.5 × 1.5 m apart. All the seedlings originated from the local natural common ash forest. The age of the infected trees ranged from 10 to 13 years, and their diameter at breast height (DBH) ranged from 7 to 10 cm. Approximately 50% of the trees were symptomatic, with bark necrosis at the stem base. The necroses quickly spread along the axis of the stem, sometimes encompassing the entire stem circumference (forming girdling cankers). Necroses usually appeared from ground level to a height of 30–50 cm. The initial color of the necrosis was light reddish to brown and subsequently turned dark brown (Figures 1A,B). These necrotic areas penetrated from the surface of the bark toward the sapwood, and the bark soon died away in these spots. Scattered perithecial stromata appeared on the surface of the dead bark (Figures 1C,D) as soon as 3–4 months from the appearance of symptoms of infection. A year after bark dieback, these areas were covered with moss or lichens (*Lecanora* sp.) on some of the stems.

### Isolation and Morphological Identification

To isolate and identify the causative agent, samples were collected from necrotic areas at the stem base (Figures 1A–C). A total of 50 trees were randomly selected for sampling, and samples of necrotic bark, approximately 0.5 × 5 × 10 cm in size, were collected using a sterilized knife or ax and transported to the laboratory. The xylem and bark fragments, 3–4 mm in size, were cut out with a scalpel sterilized in 70% ethanol and heated over an open flame. Following surface sterilization, these were plated onto Malt Extract Agar (MEA) nutrient medium (Malt Extract (Merck, Germany) and Agar (Torlak, Serbia), prepared according to the method of Booth (1971). The fragments were surface-sterilized using two methods: (a) the first method consisted of immersion of fragments in a solution of 1% sodium hypochlorite, with 4% active chlorine content, for 3 min. The fragments were subsequently washed in sterile distilled water thrice and plated onto the nutrient medium; (b) the second method consisted of immersion of fragments in a solution of 60% ethyl alcohol; next, they were briefly exposed to an open flame and then plated onto the nutrient medium. The media containing the fragments were incubated at 6°C for 48 h, then transferred to an incubator at 20°C in the dark. Following the appearance of the first hypha, they were immediately transferred onto fresh MEA media and kept for future identification tests. Fragments from all the collected samples were also plated onto an MEA media amended with Streptomycin according to Kowalski (2006) and incubated at 20°C in the dark to test whether the symptomatic tissues were positive for *Hymenoscyphus fraxineus*. In addition, fragments from necrotic tissues were plated onto a selective V8A-PARPNH media (Jung et al., 1996) and incubated at 20°C in the dark to test whether they were positive for pathogens from the *Phytophthora* genus.

To determine colony shape patterns, all the isolates were incubated at 20°C in MEA and Potato Dextrose Agar (PDA) media for 10 days and 4 weeks, respectively. The media were prepared according to the manufacturer’s instructions (HiMedia Laboratories, India), with 15 ml of media per 90 mm Petri dish. To test growth rate patterns, three replicates of five randomly selected isolates were performed on MEA and PDA media (12 ml of media per 90 mm Petri dish) and incubated for 24 h at 20°C in the dark. The isolates were then moved to 4, 10, 15, 20, 25, 27.5, 30, 32.5, 35, and 37.5°C for an additional 24 h. A cross was drawn in permanent marker at the bottom of each Petri dish, and the colony edges were defined by steel needle after every additional 24 h for 5 days, or until the colonies filled the Petri dishes completely.

Morphological observations were noted using an Olympus SZX7 stereomicroscope (Olympus Europa, Hamburg, Germany) and Ceti Magnum-T trinocular light microscope (Medline Scientific Ltd., United Kingdom). Morphological structures were recorded using a Si3000 digital camera (Fisher Scientific, United Kingdom), equipped with XliCap^®^ (Xl Imaging Ltd., United Kingdom) imaging software for the light microscope, and an EOS 3000D digital camera (Canon, Japan) for the stereomicroscope, respectively.

Identification was performed based on the characteristics of perithecial stroma, the shapes of asci and ascospores (Figures 1D–G), growth rate analyses, the colony shape patterns of pure cultures after 10 days and 4 weeks of incubation at 20°C in the dark (Figures 3A–C), and the presence of macro- (Figure 1H) and microconidia in the pure cultures.

### Molecular Identification

Two randomly selected isolates were selected for DNA extraction: one originated from *F. excelsior* in this study (NEO135), and the other originated from *Fagus sylvatica* necrotic tissue (NEO034). Selected pure cultures of these isolates were transferred onto PDA media and incubated for 2 weeks at 20°C in the dark. Total genomic DNA was isolated from the mycelial mat by scraping with a sterile scalpel from the edges of 14-days-old cultures using a DNeasy^®^ Plant Mini Kit (Qiagen, Valencia, CA, United States) according to the manufacturer’s instructions. Prior to DNA extraction, the mycelia were ground with a mortar and pestle in the presence of liquid nitrogen.

Four loci were sequenced for each isolate, namely ITS (5.8 S ribosomal DNA intervening with internal transcribed spacer 1 and 2), LSU (large subunit nuclear ribosomal DNA), *tub* (β-tubulin), and *tef1* (translation elongation factor 1-α). Amplification of the ITS locus, using primer set ITS1 and ITS4, adhered to the protocol of White et al. (1990). Amplification of the LSU locus, using primer set LSU1Fd (Crous et al., 2009) and LR5 (Vilgalys and Hester, 1990), adhered to the protocol of Quaedvlieg et al. (2012). Amplification of loci *tub*, using primer set Btub-T1 and Btub-T2 (O’Donnell and Cigelnik, 1997) and *tef1*, using primer set Tef1-728F (Carbone and Kohn, 1999) and Tef1-1567R (Rehner and Buckley, 2005), adhered to the protocols of Hirooka et al. (2013). Each reaction was performed in a 25 μl mixture containing 1 ng of genomic DNA, 0.5 μM of each primer, 0.2 μM of each dNTP, 2.5 μl of 10 × PCR reaction buffer, and 1U of DreamTaq^TM^ DNA Polymerase (Thermo Fisher Scientific). The amplification reactions were performed in a Veriti^®^ 96-Well Thermal Cycler (Applied Biosystems, Foster City, CA, United States). Immediately after the reaction, 1 μl of PCR product was electrophoresed in 1% TAE agarose gel with 1 kb DNA Ladder Plus (Invitrogen, Carlsbad, CA, United States) as a molecular weight marker. The amplified PCR fragments of DNA were purified with a CleanUp Kit (A&A Biotechnology, Gdynia, Poland). Both strands of the PCR products were sequenced using a 3730XL DNA Analyzer (Applied Biosystems, Foster City, CA, United States) at Genomed Company (Warsaw, Poland). The nucleotide sequences were read and edited using FinchTV v.1.4.0 (Geospiza Inc., Seattle, WA, United States) and deposited in the GenBank as listed in Table 1. Other sequences used in the analyses were obtained from GenBank (Table 1).

**TABLE 1:** Taxa and GenBank accession numbers used in this study.

**Neonctria species**	**GenBank accession number**			
**and isolate codes**	**ITS**	**LSU**	***tub***	***tef1*α**
*N. punicea* NEO135^*a*^	KX620770	MT379473	KX639693	MT407462
*N. punicea* NEO034^*b*^	KX620769	MT379472	KX639694	MT407461
*N. punicea* CBS 119528	KC660528	KC660602	DQ789888	KC660468
*N. punicea* CBS 134248	KC660523	KC660556	KC660703	KC660462
*N. punicea* CBS 119531	KC660507	KC660553	KC660698	KC660445
*N. punicea* CBS 242.29	KC660522	KC660565	DQ789873	DQ789730
*N. punicea* CBS 119724	KC660496	KC660568	DQ789824	KC660431
*N. coccinea* CBS 119150	KC660504	KC660577	KC660717	KC660440
*N. coccinea* CBS 118916	KC660505	KC660601	KC660719	KC660442
*N. coccinea* CBS 118914	KC660500	KC660607	KC660725	KC660435
*N. coccinea* A.R.3701	KC660502	KC660591	KC660716	KC660438
*N. hederae* CBS 125175	KC660520	KC660615	KC660760	KC660459

**^*a*^Isolated from Fraxinus excelsior in this study. ^*b*^Isolated from Fagus sylvatica in this study. A reference for the sequences used for alignment is Hirooka et al. (2013).**

**FIGURE 2:**
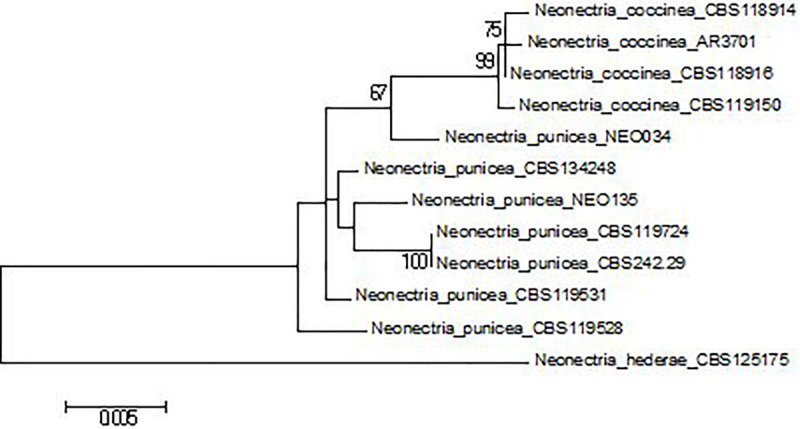
Multilocus phylogenetic tree (Maximum Likelihood) of *Neonectria* spp. based on the Tamura-Nei model with the best log likelihood (–4609.9001). Bootstrap values ≥ 70%. The tree is drawn to scale, with branch lengths measured in the number of substitutions per site.

Sequence data were initially aligned in CLC Main Workbench 8. Following sequence alignment, a Maximum Likelihood (ML) tree was estimated for each DNA region, and the multilocus data set (a total of 2,628 bp; positions containing gaps were eliminated). For each sequence set, the best-fitting substitution model was selected. The evolutionary history was inferred using the Maximum Likelihood method based on the Jukes-Cantor (ITS), Kimura 2-parameter (LSU, *tef1*), Tamura 3-parameter (*tub*), and Tamura-Nei (multilocus set) models. Initial tree(s) for the heuristic search were obtained automatically by applying Neighbor-Join and BioNJ algorithms to a matrix of pairwise distances estimated using the Maximum Composite Likelihood (MCL) approach and then selecting the topology with superior log likelihood value. Representative GenBank sequences of *Neonectria punicea*, *N. coccinea*, and *N. hederae* (outgroup) were included (Table 1) for confirmation of morphological identification (Figure 2). Bootstrap (BP) analyses were replicated 1,000 times. A reciprocal 70% BP threshold was used to determine if partitions could be combined into a single phylogeny.

### Pathogenicity of *N. punicea*

**FIGURE 3:**
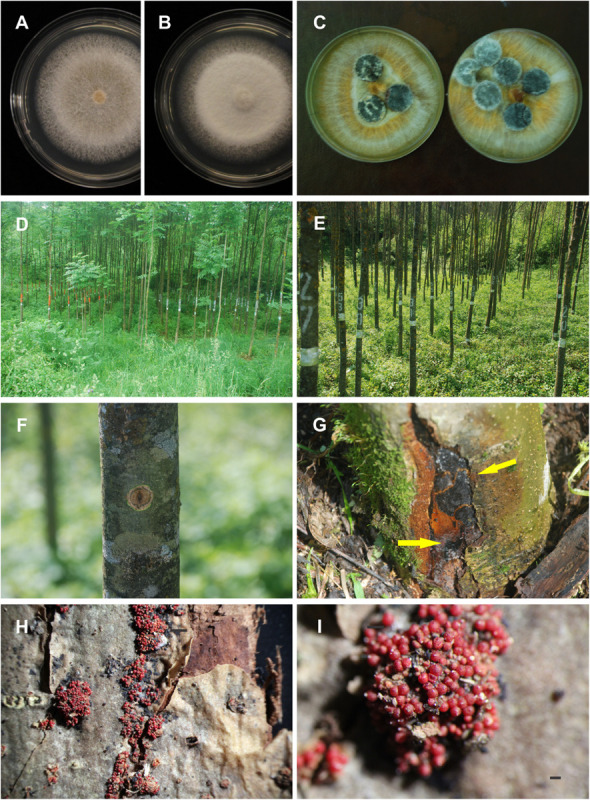
Colony shape patterns and pathogenicity of *Neonectria punicea*. **(A)** Pure culture on MEA, and **(B)** on PDA media after 10 days of incubation at 20°C in the dark. **(C)** Sterilized ash wood fragments plated on the surface of pure cultures to be overgrown by *N. punicea* colonies. **(D,E)** Stand where inoculation was carried out. **(F)** Necrosis on ash trees 12 months post inoculation at BH. **(G)** Ash trees 12 months post inoculation at SB with *N. punicea* originating from common ash (arrows indicate the position of perithecial stromata). **(H)** Numerous stromata with fruiting bodies. **(I)** Close view of stromata with numerous perithecia. Scale bars: **(I)** = 200 μm.

One isolate of *N. punicea* (NEO135), obtained from the necrotic tissue of juvenile *F. excelsior*, was randomly selected and used to test the pathogenicity of the isolated fungus. As a comparison, an unusual isolate of *N. punicea* (NEO034), obtained from the necrotic tissue of mature *F. sylvatica*, was developed and used in a pathogenicity test. This isolate differed from the other *N. punicea* isolates as the macroconidia were not formed as pustules in older colonies. Although they had the same cardinal temperatures on MEA and PDA, their growth rates were slightly lower, averaging 2.83 mm per day at 25°C on MEA and 2.28 mm per day at an optimal 20°C on PDA. Both isolates were developed on MEA media and incubated at 20°C in the dark for 4 weeks until they filled the 90 mm Petri dish. Wood fragments, 4–5 mm high and 9 mm in diameter, were subsequently collected from living, healthy, common ash branches with a metal cork borer of the same size and autoclaved at 120°C for 20 min. After cooling, the wood fragments were placed wood-side down on the developed mycelia of both isolates and incubated for an additional 4 weeks (Figure 3C).

Artificial inoculation of 180 stems of 10–12-years-old *F. excelsior* (in three repetitions) was performed in the field (Figures 3D,E) using the underbark stem inoculation test (Karadžić et al., 2019). A total of 60 trees (30 with wounded tissues and 30 unwounded ones) were used for both inoculation with the isolate from *F. excelsior* and as the control. Furthermore, 60 trees were inoculated with the isolate originating from *F. sylvatica* as a comparison.

In the case of the isolate originating from *F. excelsior*, wounds were made on 30 stems (10 stems per repetition) using a metal cork borer (9 mm in diameter). The inoculum overgrown with fungal mycelium was subsequently applied to the injured sites in a wood to wood position. The inoculation was performed at two sites on the same tree, including the stem base (SB) (approximately 0.2 m) and breast height (BH) (approximately 1.30 m). The inoculum containing the fungus was applied directly to the bark without previous wounding on 30 stems (at the SB and BH sites). The remaining 30 stems used as a control group were wounded with a cork borer, and sterile wood fragments were retrieved. The same procedure was similarly applied for the inoculation of 90 *F. excelsior* trees aged between 10 and 12 years with the isolate from *F. sylvatica*. After inoculation, the inoculated sites were covered with a small piece of sterile cotton and sealed with plastic sheeting. The average DBH in the group of trees inoculated with the isolate from *F. excelsior* was 7.5 ± 0.17 cm, whereas DBH in the group of trees inoculated with the isolate from *F. sylvatica* was 6.7 ± 0.22 cm.

Inoculated trees were first inspected after 12 months, and control reisolations from 50% of randomly selected trees within all the groups were performed. The experiment was completed after 24 months, and reisolations were performed at the edges of the necrotic zones and from the formed fruiting bodies.

### Scanning Electron Microscopy (SEM)

Wood sections (transverse, radial, and tangential surfaces), sampled from three infected trees, were mounted on specimen stubs, sputter-coated with gold in the Sputter Coater K650X (Quorum Technologies, Ashford, United Kingdom) in an argon atmosphere, and examined by high-vacuum SEM using a JEOL JSM-6390 instrument (JEOL, Tokyo, Japan) operating at 15 kV.

### X-ray Micro-Computed Tomography

X-ray micro-computed tomography (X-ray microCT) imaging of woody tissues sampled from three infected trees was carried out with a Phoenix V|Tome|X L 240 device (GE Sensing & Inspection Technologies, Wunstorf, Germany) equipped with a 180 kV/15 W high-power nanofocus X-ray tube. Scanning parameters were set as follows: voltage 70 kV; current 220 μA; projections 1,800; average 3, skip 1, timing 500 ms; and voxel size 2.5 μm. After the scanning process was completed, three-dimensional data sets were evaluated using VGSTUDIO MAX 2.2 software for industrial CT data (Volume Graphics, Heidelberg, Germany).

### Statistical Analyses

The surface of each necrotic area was calculated using the mathematical formula for elliptic surfaces based on length and width. Analysis of variance was performed using a Generalized Linear Model (GLM), with necrotic area height, width, and surface as dependent variables; the site of inoculation (SB or BH), time after inoculation, and origin of inoculum as predictors; and plant diameter as a covariate (α = 0.05). The significance of differences in mean necrotic area length, width, and surface between different treatments was tested using the Duncan *post hoc* test (α = 0.05). Statistical procedures were performed using STATISTICA 13.4 (TIBCO software Inc. 1984–2018).

## Results

### Isolation and Morphological Identification

After the isolation tests, 100% of the plated pieces were positive, and 54 isolates were obtained. There was no difference between surface sterilization methods, and both proved to be efficient during isolation. The mycelium growing on MEA and PDA media gradually assumed a sparse, hairy appearance (Figures 3A,B). In the MEA media, it was mostly aerial surrounding the inocula, whereas, in PDA, it was slightly cottony and located in the middle of the colony. After 10 days of incubation at 20°C in the dark, the colonies on both tested media were white (Figures 3A,B), whereas the older, aerial hyphae were cottony whitish-yellow or light reddish-brown; the agar also turned reddish-brown (Figure 3C). Colony growth was medium-fast, and the mycelium grew under a range of temperatures from 4 to 30°C. The optimum temperature for growth in the MEA medium was 25°C, with an average growth rate of 3.19 ± 0.05 mm per day. On the contrary, that of the PDA medium was 20°C, with an average growth rate of 3.25 ± 0.06 mm per day. After 7 days of incubation, microconidia appeared in cultures incubated at 20°C in the dark. These were elliptical to cylindrical, formed on lateral phialides, and were single-celled or two-celled, reaching dimensions of 8.6 ± 0.2 × 3.2 ± 0.07 μm, with a range of 6.6–12.3 × 2–4 μm (*N* = 47). In cultures incubated for more than 4 weeks, macroconidia were formed as small, light-yellow pustules, scattered mostly in the growth center. The macroconidia were cylindrocarpon-like, hyaline, cylindrical, narrowing slightly toward the rounded ends and with three–eight septa (Figure 1H). The average dimensions were 47.5 ± 1 × 6.6 ± 0.08 μm, with a range of 36.1–55.7 × 5.7–7.5 μm (N = 34). However, none of the examples yielded *Hymenoscyphus fraxineus* or *Phytophthora* spp. colonies.

The perithecia on the necrotic bark were densely crowded and developed in succession on an erumpent reddish stroma (Figure 1D) formed of homogeneous, globose, thin-walled cells. The stroma base penetrated the lenticels and was usually attached to the cortical tissue of the host by this narrow wedge of tissue (Figure 1E). Perithecia were red to reddish-brown, globose, subglobose to ovate, with a small apical disc (ostiolum), and averaged 281.8 ± 9.5 × 265 ± 8.6 μm, with a range of 205.5–342.4 × 185.6 × 313.8 μm. Asci were clavate (Figure 1F), with a rounded apex and eight monostichous to apically distichous ascospores and averaged 88 ± 2.8 × 9 ± 0.28 μm in our measurements, with a range of 73.3–117.2 × 7.4–11.4 μm. Ascospores were hyaline to light brown at maturity with slightly roughened walls, fusoid, broadly fusoid to ellipsoid, and slightly constricted at the single central septum (Figure 1G). The average dimensions of the ascospores were 12.5 ± 0.2 × 5.2 ± 0.07 μm, with a range of 9–16.2 × 3.7–6.3 μm. The ascospores usually showed variation in the size and shape of each ascus.

### Molecular Identification

DNA sequences of four loci, i.e., ITS (446 bp), LSU (797 bp), *tub* (approximately 590 bp), and *tef1*α (approximately 800 bp), were successfully obtained for both isolates of *N. punicea*. Based on the alignment of the ITS sequences to the GenBank database, both of the isolates belong to the *N. punicea* taxon. Further analysis of the remaining loci suggests ambiguous species affiliation of isolate NEO034. Generally speaking, two main patterns of tree topology were found. Based on ITS and LSU analyses (Supplementary Figures S1, S2), both of the isolates belonged to *N. punicea* clade (ML BP > 50%), whereas *tef1* and *tub* analyses (Supplementary Figures S3, S4) showed that isolate NEO034 is more congenial with *N. coccinea.* A similar result, well supported by BP values, was obtained by the analysis of concatenated multilocus sequence (ML BP > 90%) (Figure 2) and suggested that isolate NEO034, originating from *F. sylvatica*, does not belong to *N. punicea* clade.

### Pathogenicity of *N. punicea*

**FIGURE 4:**
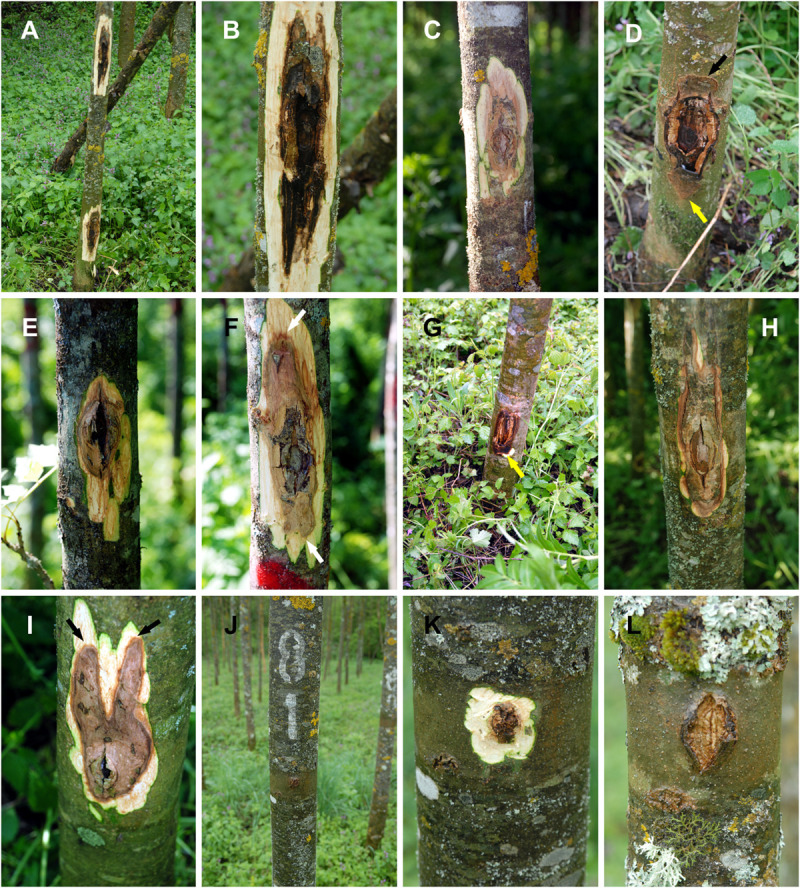
Representative photos of ash stems 24-months post inoculation. *Neonectria punicea* originating from common ash: **(A)** Tree inoculated at both SB and BH. **(B,C)** Cankers at BH. **(D)** Canker at SB, with sunken bark (black arrow) and active margin (yellow arrow). *N. punicea* originating from European beech: **(E,F)** Cankers at BH with active margin (white arrows). **(G)** canker at SB with active margin (yellow arrow). **(H)** Irregular shape of canker at BH. **(I)** Irregular shape of canker at SB with active margins (black arrows). **(J–L)** Control trees.

After pathogenicity testing in the field, both isolates of *N. punicea* were found to be pathogenic to ash trees in the case of previous wounding of the bark (Figures 3, 4). However, no wood tissue infections occurred in direct inoculum application to the healthy bark or the control group. After 12 months, the fungus *N. punicea* was reisolated from stems inoculated with the isolate from *F. excelsior* (Figure 3F) in 94% of the cases. However, in stems infected with the isolate originating from *F. sylvatica* the fungus was reisolated in 67% of the cases. The control trees remained without developing necroses. In contrast, inoculated trees showed necrotic areas of different sizes, which were visible at both BH and SB sites (Figure 3G), and the formation of numerous stromata with fruiting bodies was recorded at the inoculation sites (Figures 3H,I).

Twenty-four months after inoculation, all the trees within the inoculated groups showed visible cankers of different sizes, and the experiment was completed. Apart from the slight yellowing and atrophy of the leaves in some cases, other symptoms, such as dieback of crowns and branches, were not recorded. Reisolation was successful from the cankers of all the inoculated trees (100%), whereas reisolation from the control group was negative. The necrotic areas on the stems spread mostly in a regular shape, initially forming a circle around the inoculation site (Figure 3F), subsequently forming symmetric or asymmetric elliptic shapes (Figures 4A–G). Some irregular and interesting shapes were also recorded (Figures 4H,I). The control trees remained without visible cankers or necroses and the wound sites were almost completely closed over by callus tissue (Figures 4J–L). Typical sunken bark was recorded on the lesions in most of the cases.

The substantial impact of time post-inoculation (T), inoculum origin, place of inoculation, and interactions between T and inoculum origin, as well as covariates, were established, but not in relation to interactions between T, inoculum origin, and site of inoculation (Table 2).

**TABLE 2:** Results of multivariate tests of significance.

**Source of variation**	**Effect (df)**	**Error (df)**	***F***	***p***
Time post inoculation [T]	**3**	**465**	**85.88**	**0.0000**
Inoculum origin (IO)	**6**	**930**	**99.51**	**0.0000**
Place of inoculation (PI)	**3**	**465**	**4.62**	**0.0034**
T × IO	**6**	**930**	**13.12**	**0.0000**
T × PI	**3**	**465**	**3.26**	**0.0215**
IO × PI	**6**	**930**	**3.96**	**0.0006**
T × IO × PI	6	930	1.56	0.1542
Covariate [D_1.3_]	**3**	**465**	**2.87**	**0.0362**

**F-ratios are shown. Significant P-values are indicated in bold. Impact shown in bold is statistically significant.**

The substantial impact of T and inoculum origin on necrosis length, width, and the surface was also established (Table 3). Interactions between T and inoculum origin also had a significant impact on all necrosis traits.

**TABLE 3:** Results of general linear models for analysis of the spread of *N. punicea* necroses, including individual stem diameter at breast height as a covariate.

**Source of variation**	**d.f.**	**Necrosis length**		**Necrosis width**		**Necrosis surface**	
		***F***	***p***	***F***	***p***	***F***	***p***
Time post inoculation (T)	1	**103.21**	**0.0000**	**193.47**	**0.0000**	**95.65**	**0.0000**
Inoculum origin (IO)	2	**141.85**	**0.0000**	**152.74**	**0.0000**	**77.64**	**0.0000**
Place of inoculation (PI)	1	0.00	0.9852	**5.57**	**0.0187**	1.76	0.1851
T × IO	2	**24.82**	**0.0000**	**31.29**	**0.0000**	**24.24**	**0.0000**
T × PI	1	0.17	0.6843	2.37	0.1242	0.54	0.4641
IO × PI	2	0.14	0.8736	**5.26**	**0.0055**	1.22	0.2955
T × IO × PI	2	0.25	0.7767	0.97	0.3790	0.44	0.6459
Covariate [D_1.3_]	1	0.68	0.4088	**7.00**	**0.0084**	2.40	0.1223
Error	467						

**Significant P-values are indicated in bold.**

**FIGURE 5:**
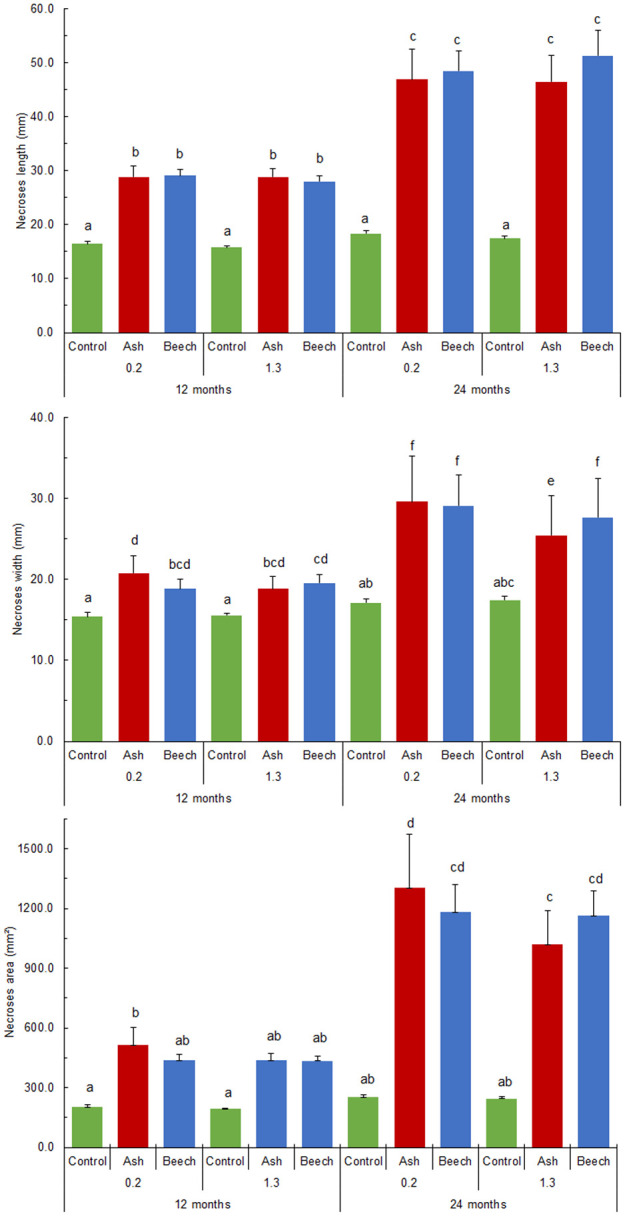
*N. punicea* necrosis spreading axially **(A)**, tangentially **(B)** and necrosis surface **(C)** 12 and 24 months after inoculation by inoculum originating from common ash and European beech. Means (±SE) under different letters above, are significantly different based on the Duncan *post hoc* test at *p* < 0.05.

Twelve months post-inoculation, necrotic area lengths and widths at SB were statistically significantly larger compared to the control trees in the case of both the used isolates, whereas only the isolate from common ash caused significantly larger necrotic surfaces (Figure 5). No significant difference was recorded between the used isolates (Figure 5). A similar situation was recorded at the site of BH inoculation, where both isolates did not statistically significantly differ from the control (Figure 5) in the case of necrotic surfaces.

Twenty-four months post-inoculation, statistically significantly larger necrotic area lengths, widths, and surfaces (compared to the control trees) were recorded in the case of both tested isolates (Figure 5). Although not significantly different, there was a slight trend of greater widths and necrotic surfaces caused by the isolate from *F. excelsior* recorded at SB 24-months post-inoculation (Figure 5). A trend of larger necrotic areas caused by the isolate from *F. sylvatica* at BH was recorded, in contrast, and necrotic area widths were statistically significantly greater.

When comparing necrotic area size and time post-inoculation, all the necrosis values increased significantly within 12 months between the two measurements. Specifically, necrotic area lengths at SB and BH increased 1.6 times in the case of the isolate from *F. excelsior*, and 1.7 and 1.8 times in the case of the isolate from *F. sylvatica* (Figure 5). A similar situation was recorded in the case of necrotic area widths, and the situation in the case of necrotic area surfaces was even more pronounced. Necrotic area surfaces at SB and BH increased from 515.2 ± 88.16 to 1305.4 ± 267.92 mm^2^, and from 438.2 ± 31.65 to 1020.5 ± 167.36 mm^2^, respectively, in the case of tested isolate originating from *F. excelsior*. Necrotic area surfaces at SB and BH in the case of the isolate from *F. sylvatica* also increased from 437.6 ± 27.65 to 1182.5 ± 138.54, and 435.1 ± 23.88 to 1163.7 ± 126.81 mm^2^, respectively.

### Tree Responses and the Spread of Infection

**FIGURE 6:**
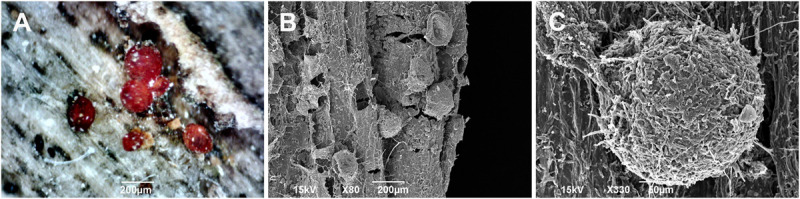
Light microscopy **(A)** and scanning electron microscopy **(B,C)** images of the globose perithecia of *Neonectria punicea* isolate NEO135 growing on the wood of an infected *Fraxinus excelsior* tree. Scale bars: **(A,B)** = 200 μm, **(C)** = 50 μm.

Upon inoculation, the fungal mycelium colonized the surface surrounding the inoculation sites. This was followed by rupture of the bark and cambium necrosis. The inoculation sites became extensive open cankers with necrotic tissue. Infected trees responded by forming a small concentric annual growth ring of the canker in the next growing season after inoculation. However, a dysfunction in the discontinuous and necrotic cambium in close proximity to the inoculation sites prevented the spread of the callus tissue of the concentric annual growth ring throughout the cankers. The same situation was repeated during the second growing season after inoculation. However, in the third year after inoculation, a large area of the concentric annual growth ring tissue was formed, which began to close over the cankers. In addition, small, red, and globose perithecia were formed at the necrotic sites of both the first and second annual growth rings after inoculation (Figures 6A–C).

**FIGURE 7:**
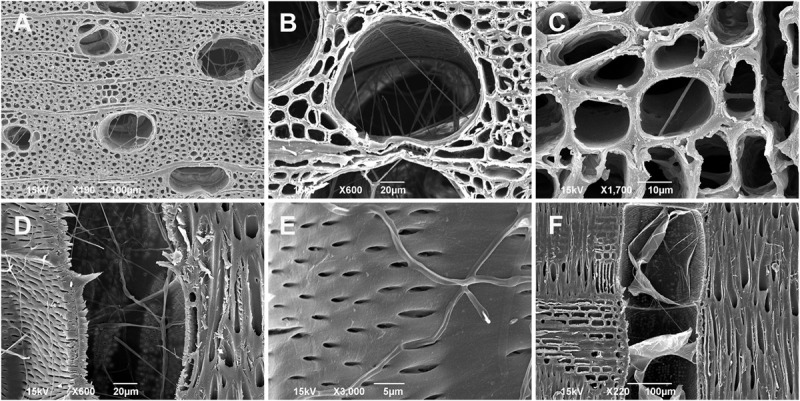
Scanning electron microscopy images of juvenile *Fraxinus excelsior* wood (the outermost annual growth ring) infected by *Neonectria punicea* isolate NEO135. **(A,B)** Growth of fungal hyphae inside earlywood vessels, cross-sections. **(C)** Spread of hyphae through axial parenchyma cells, cross-section. **(D)** Hyphae growing inside earlywood vessel, radial section. **(E)** Hyphae penetrating through pits and invading the earlywood vessel, radial section. **(F)** Tyloses formed in an earlywood vessel in response to fungal inoculation, radial section. Scale bars: **(A,F)** = 100 μm, **(B,D)** = 20 μm, **(C)** = 10 μm, **(E)** = 5 μm.

Regarding the fungal spread through the xylem tissues, the hyphae penetrated mostly into the outermost annual growth ring. The majority of hyphae were found in the large earlywood vessels and rarely in the axial parenchyma cells (Figures 7A–E and Supplementary Video S1). Infected trees responded with the formation of tyloses in vessels to prevent a rapid fungal spread through the axial vascular transport pathway (Figure 7F and Supplementary Video S2).

## Discussion

The presence of *N. punicea* in *F. excelsior* was confirmed by isolation, the morphological features of the micro- and macroconidia, perithecia, asci, and ascospores, the shape and growth rates of the obtained isolates, and their molecular identification. This was the only isolated organism from the margins of active collar cankers in the studied locality. To our knowledge, this is the first report of *N. punicea* on common ash trees in B&H. *N. punicea* has a wide host range, and it was previously reported in multiple hosts in Europe, Asia, and North America (Booth, 1959; Hirooka et al., 2013; Langer, 2017). This fungus has also been reported in common ash in Germany (Langer, 2017; Meyn et al., 2019) and recently in the narrow-leaved ash in neighboring Croatia (Kranjec Orlović et al., 2020). In B&H, *N. punicea* (*Nectria punicea* var. *ilicis* Booth) was previously mentioned in studies on species from the *Neonectria* genus in beech forests (Lazarev and Jokanović, 2007), and this could be considered as the first report of *N. punicea* in B&H. Therefore, it is most likely that *N. punicea* has been present for an extended period in B&H but has not been studied in detail to date. In contrast, *N. coccinea* (Pers.: Fr.) Rossman and Samuels, the cause of BBD, is widely present in beech trees in B&H (Lazarev and Jokanović, 2007; Karadžić et al., 2012b), causing substantial damage in different beech ecosystems along with other *Neonectria/Nectria* species and other damaging agents.

Despite the attempts to isolate *H. fraxineus* from the recorded necrotic tissues, all the collected samples remained negative for this pathogenic fungus. In studies by Stanivuković et al. (2014), *H. fraxineus* was recorded near the studied planted forest, but only in plants newly introduced from another area, while local seedling stock and plants from native natural regeneration remained completely free of ash dieback symptoms. Ash dieback fungus was introduced into the planted forest with tree seedling stock that had been previously infected; however, local, native plants showed some level of resistance and tolerance to *H. fraxineus* infection as reported previously in several studies (Stener, 2013, 2018; Lobo et al., 2014; McKinney et al., 2014). It is also possible that the inoculum level was not yet high enough to cause any significant symptoms or to be detected. In this scenario, *N. punicea* originating from various surrounding hosts could colonize these weakened plants and cause some secondary symptoms, as demonstrated by Meyn et al. (2019). However, ash trees originating by natural regeneration in the nearby forest of the surrounding area did not show any signs of collar necrosis or *H. fraxineus* presence. Intriguingly, these surrounding plants are of similar age and are exposed to similar environmental conditions and, subsequently, a similar inoculum level of *H. fraxineus* and *N. punicea*. The presence of necrotic tissues on newly introduced plants could be partially explained by additional stress due to planting and moving them onto post-agricultural land. This phenomenon was recorded in the case of beech trees, where inoculated fresh beech logs were more susceptible to the same fungal isolates as inoculated living trees (Kunca and Leontovyč, 1999). Ash dieback symptoms were also not recorded in the crowns and branches of trees affected with collar cankers in this planted forest, suggesting the absence of *H. fraxineus*. However, similar situations where only symptoms such as collar cankers appeared were previously recorded (Muñoz et al., 2016; Enderle et al., 2017). Additional studies on this subject, as well as studies at other localities with similar symptoms, are needed to determine the presence of *H. fraxineus*, as demonstrated by Meyn et al. (2019). Regardless of the causative agent, the site conditions in this planted forest are favorable for collar necrosis development based on the descriptions of similar sites (Marçais et al., 2016; Enderle et al., 2017).

Following a pathogenicity test in field conditions, all injured stems, which were subsequently inoculated with wood fragments overgrown by mycelia, were infected. It was found that the fungus spreads more rapidly in the axial, rather than the radial direction on the stems (Figure 5). However, almost no statistically significant difference was established between inoculation at SB and BH (Figure 5). Although inoculated at two sites, with significantly larger necroses being recorded (Figures 3, 4), the plants did not show signs of severe decline for over 2 years of observation, except for a few slight symptoms in the crowns. A similar situation also continued in the third year, post-inoculation. This could be explained by the duration of observation, and the infection process and pathogenesis itself are certainly variable under natural conditions. The exact time of contamination and pathogen penetration into host tissues is also very hard to determine under natural conditions. In the case of common ash and *H. fraxineus* infections via ascospores, some findings were obtained previously (Cleary et al., 2013; Mansfield et al., 2018). Additional studies are needed to clarify these issues and determine the schedule of development in ash trees in the case of *N. punicea*.

In most of the underbark pathogenicity trials, under laboratory and field conditions, one inoculation site is usually created in the selected stem position (SB or BH) (Karadžić et al., 2019; Vemić et al., 2019) using a sterilized scalpel, metal cork borer, or similar tool (Biggs, 1992). Contrary to this, in the case of natural infection, there are usually several infection points, which means that plants need to allocate significant resources for their defense. In the case of *H. fraxineus* infection of common ash trees, it has been proven through genotyping studies that in addition to several different infection sites, there are also several different genotypes infecting the same host tree (Husson et al., 2012; Meyn et al., 2019), causing the tree to weaken substantially and eventually decline. No genotyping studies of *N. punicea* in infected common ash tissue have been carried out to date, and it would be interesting to perform these studies in the future and test the aggressiveness of different genotypes of both *H. fraxineus* and *N. punicea*. The genome sequence for *N. punicea* has also been recently studied and published (Salgado-Salazar and Crouch, 2019), making future development of detection methods and working with this pathogen easier.

Another possible scenario is that *H. fraxineus* is not present in the studied common ash plantation and that *N. punicea* infection was achieved through wounds and natural openings, such as lenticels. After the pathogenicity trial, the results showed that none of the trees without previously damaged bark, to which the inocula were applied, were infected. Similar results were obtained from the studies on *N. coccinea* development in European beech in Slovakia (Kunca, 2005) and in studies on *Cryphonectria parasitica* (Murrill) Barr pathogenicity on sessile oak trees in Serbia (Karadžić et al., 2019). This indicates that the fungus is unable to infect stems with undamaged bark, which corresponds to the known *Nectria/Neonectria* mechanisms of infection (Lortie, 1969; Perrin, 1977; Marinković and Karadžić, 1985; Biggs, 1992; Karadžić et al., 2012a). However, cases when some species from the *Nectria*/*Neonectra* genus were able to penetrate the healthy bark and cause infections, as demonstrated by Lortie (1969), have also been recorded. Therefore, it can be concluded that the *N. punicea* fungus most likely has no enzymes that can disintegrate the dead cork layer of the host bark. Bark injuries (caused by insects, frost, wind-induced cracks at the stem base, mechanical damage caused by animals or during harvesting and hauling of trees, etc.), or natural openings such as lenticels, are therefore favorable entry points for ascospores transported by raindrops, where they can start germinating and establish an infection. The studied area is exposed to low temperatures (Supplementary Table S1) and frosts, which create microcracks in the bark, and these are also favorable points of entry for *N. punicea* and the other opportunistic pathogens. Lenticels have been previously suggested as infection points on ash shoots (Nemesio-Gorriz et al., 2019) and ash collars (Meyn et al., 2019). The *N. punicea* var. *ilicis* species description (Booth, 1959) describes the stromata as having a wedge-shaped base and appearing from the lenticels. We obtained similar findings during pathogen identification (Figure 1E), and together with the results from path trials, this suggests that the lenticels at the stem base are the most probable point of entry for *N. punicea*. It may be appropriate to perform future detailed pathogenicity tests using ascospore suspensions to clarify these conclusions and previous assumptions.

Additionally, because the stem base is covered by surrounding weeds, ground flora, and sometimes even moss, light deficiency and moisture conditions in these areas are optimal for spore germination and the establishment of an infection. This can also explain why most infections by *N. punicea* and *H. fraxineus* (Meyn et al., 2019) originate at the root collar level. Likewise, a similar phenomenon was observed in natural beech stands affected by BBD in B&H and Serbia (Karadžić, unpublished data).

SEM and X-ray microCT imaging shed more light on how the hyphae of *N. punicea* penetrate into woody tissues when living bark tissues, including phloem and cambium, are decaying. Infected trees responded with the formation of tyloses in vessels to prevent fungal spread through the axial vascular transport pathway. Similar defense mechanisms were also previously reported in interspecific *Ulmus* hybrids infected by *Ophiostoma novo-ulmi* Brasier, a causative agent of Dutch elm disease (Ďurkovič et al., 2014).

During this study, an unusual isolate (NEO034) of *N. punicea* originating from mature *F. sylvatica* tissue was used to compare the pathogenicity trial. Based on morphological characteristics, this isolate was preliminarily identified as *N. punicea* what was confirmed by the sequencing of ITS and LSU loci (Supplementary Figures S1, S2). After further analyses, based on the sequencing of *tef1* and *tub* loci (Supplementary Figures S3, S4) and multilocus sequence alignment (Figure 2), the NEO034 isolate originating from *Fagus sylvatica* does not belong to the *N. punicea* clade and it is closer to *N. coccinea*. However, this isolate is also clearly different from *N. coccinea* by having different colony shape patterns and lower maximal temperature for growth and does not grow at 32.5°C on tested MEA and PDA media. This indicates either the presence of a new species or some hybrid from the *Neonectria* genus on *F. sylvatica* trees affected by BBD, and additional studies are required for a final description.

Nevertheless, this isolate was equally aggressive, and after 24 months of incubation, it demonstrated a trend of greater necrotic area lengths at BH compared to the isolate from *F. excelsior*, whereas the necrotic area widths were statistically significantly longer (Figure 5). This implies a high risk for ash stands in the future due to the presence of huge inocula reservoirs from *F. sylvatica* trees affected by BBD. It would be interesting to also test the aggressiveness of *N. coccinea* isolates as a causative agent of BBD on ash plants and the aggressiveness of *N. punicea* originating from *F. excelsior* on *F. sylvatica* plants to clarify these potential risks and define appropriate management strategies.

The ability of *N. punicea* to cause lesions and discoloration in *F. excelsior* tissues was demonstrated for the first time in this study. Screening and selection of common ash genotypes resistant to *H. fraxineus* were suggested to be possible (Stener, 2018) and will be the main task of future preservation and breeding programs for this important tree species (Pautasso et al., 2013). This study results suggest that there is a serious threat to both planted and naturally regenerated forests, particularly to the long-term resistance screening program of *F. excelsior*. Therefore, this pathogen, among others, should be taken into consideration and integrated within future ash resistance and breeding programs.

## Data Availability Statement

The datasets presented in this study can be found in online repositories. The names of the repository/repositories and accession number(s) can be found in the article/Supplementary Material.

## Author Contributions

DK and IM: conceptualization. DK, IM, SM, KS, and JĎ: data curation and writing—original draft. DK, ZS, IM, SM, and ZR: methodology—sampling, isolation, morphological identification, and methodology—pathogenicity test. KS: methodology—molecular identification. JĎ, VR, and MK: methodology—SEM and X-ray microCT. DK, IM, JĎ, and SM: writing—review and editing. All authors contributed to the article and approved the submitted version.

## Conflict of Interest

The authors declare that the research was conducted in the absence of any commercial or financial relationships that could be construed as a potential conflict of interest.

## Supplementary Material

Supplementary Figure 1 Phylogenetic analyses of *Neonectria* spp. isolates by the Maximum Likelihood method based on the ITS sequences. The tree with the highest log likelihood (−770.6076) is shown. Bootstrap values ≥ 70%. The percentage of trees in which the associated taxa are clustered together is shown next to the branches. The tree is drawn to scale, with branch lengths measured in the number of substitutions per site.

Supplementary Figure 2 Phylogenetic analyses of *Neonectria* spp. isolates by the Maximum Likelihood method based on the LSU sequences. The tree with the highest log likelihood (−1192.6684) is shown. Bootstrap values ≥ 70%. The percentage of trees in which the associated taxa are clustered together is shown next to the branches. The tree is drawn to scale, with branch lengths measured in the number of substitutions per site.

Supplementary Figure 3 Phylogenetic analyses of *Neonectria* spp. isolates by the Maximum Likelihood method based on the *tef1* sequences. The tree with the highest log likelihood (−1440.5161) is shown. Bootstrap values ≥ 70%. The percentage of trees in which the associated taxa are clustered together is shown next to the branches. The tree is drawn to scale, with branch lengths measured in the number of substitutions per site.

Supplementary Figure 4 Phylogenetic analyses of *Neonectria* spp. isolates by the Maximum Likelihood method based on the *tub* sequences. The tree with the highest log likelihood (−1052.3578) is shown. Bootstrap values ≥ 70%. The percentage of trees in which the associated taxa are clustered together is shown next to the branches. The tree is drawn to scale, with branch lengths measured in the number of substitutions per site.

Supplementary Video 1 Spatial X-ray micro-computed tomography imaging of a vessel. The video shows the presence of hyphae inside the vessel (the area is marked with a red ellipse).

Supplementary Video 2 Spatial X-ray micro-computed tomography imaging of tyloses. The video shows tyloses filling and plugging the lumen of an earlywood vessel in response to fungal inoculation.
