# Supplementary figures and images for: Development of Neonectria punicea Pathogenic Symptoms in Juvenile Fraxinus excelsior Trees
Source: Front Plant Sci. 2020 Dec 23;11:592260. doi: 10.3389/fpls.2020.592260 (PMC7785714; doi:10.3389/fpls.2020.592260)

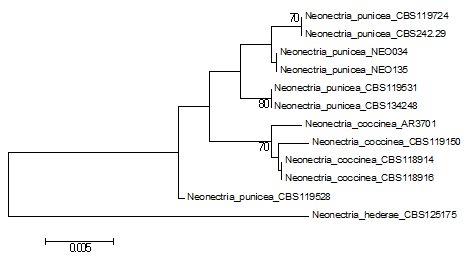

Supplement: Supplementary Figure 1 — Phylogenetic analyses of Neonectria spp. isolates by the Maximum Likelihood method based on the ITS sequences. The tree with the highest log likelihood (−770.6076) is shown. Bootstrap values ≥ 70%. The percentage of trees in which the associated taxa are clustered together is shown next to the branches. The tree is drawn to scale, with branch lengths measured in the number of substitutions per site. [file Image_1.JPEG]

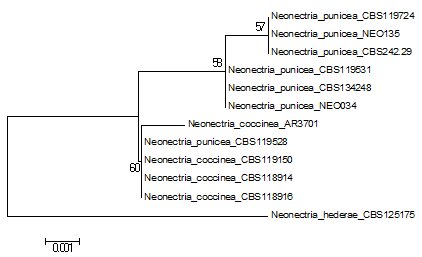

Supplement: Supplementary Figure 2 — Phylogenetic analyses of Neonectria spp. isolates by the Maximum Likelihood method based on the LSU sequences. The tree with the highest log likelihood (−1192.6684) is shown. Bootstrap values ≥ 70%. The percentage of trees in which the associated taxa are clustered together is shown next to the branches. The tree is drawn to scale, with branch lengths measured in the number of substitutions per site. [file Image_2.JPEG]

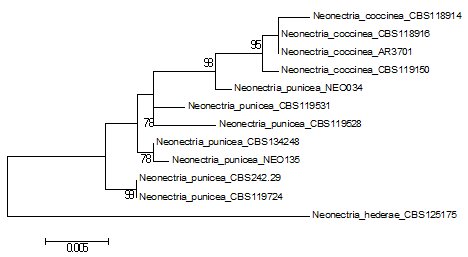

Supplement: Supplementary Figure 3 — Phylogenetic analyses of Neonectria spp. isolates by the Maximum Likelihood method based on the tef1 sequences. The tree with the highest log likelihood (−1440.5161) is shown. Bootstrap values ≥ 70%. The percentage of trees in which the associated taxa are clustered together is shown next to the branches. The tree is drawn to scale, with branch lengths measured in the number of substitutions per site. [file Image_3.JPEG]

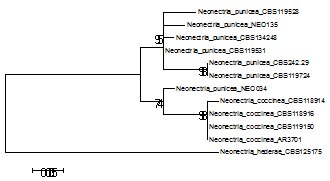

Supplement: Supplementary Figure 4 — Phylogenetic analyses of Neonectria spp. isolates by the Maximum Likelihood method based on the tub sequences. The tree with the highest log likelihood (−1052.3578) is shown. Bootstrap values ≥ 70%. The percentage of trees in which the associated taxa are clustered together is shown next to the branches. The tree is drawn to scale, with branch lengths measured in the number of substitutions per site. [file Image_4.JPEG]
