# Supplementary material for: Development of Neonectria punicea Pathogenic Symptoms in Juvenile Fraxinus excelsior Trees
Source: Front Plant Sci. 2020 Dec 23;11:592260. doi: 10.3389/fpls.2020.592260 (PMC7785714; doi:10.3389/fpls.2020.592260)
Supplement: Supplementary file 7 [file Table_1.DOCX]

**Supplementary Table S1.** Average monthly temperatures at the studied locality obtained from the TFA® NEXUS weather station for the 2010-2019 period.

| **Months** | **Years** | | | | | | | | | | **Average** |
| --- | --- | --- | --- | --- | --- | --- | --- | --- | --- | --- | --- |
|  | **2010** | **2011** | **2012** | **2013** | **2014** | **2015** | **2016** | **2017** | **2018** | **2019** |  |
| I | -1 | 0.0 | 0.2 | 1.6 | 5.0 | 2.0 | 1.2 | -6.3 | 3.8 | -1.7 | **0.5** |
| II | 0.1 | -1.5 | -6.0 | -0.1 | 5.4 | -0.9 | 6.3 | 4.0 | -2.4 | 3.0 | **0.8** |
| III | 4.8 | 4.1 | 6.1 | 3.6 | 7.2 | 4.6 | 5.6 | 7.2 | 2.7 | 7.2 | **5.3** |
| IV | 9.2 | 9.6 | 10.4 | 10.7 | 10.4 | 9.3 | 11.4 | 9.1 | 13.1 | 9.6 | **10.3** |
| V | 13.9 | 12.8 | 13.5 | 13.8 | 13.3 | 15.0 | 13.6 | 14.6 | 15.7 | 11.2 | **13.7** |
| VI | 17.4 | 17.5 | 20.0 | 17.2 | 17.4 | 17.6 | 18.6 | 19.8 | 17.5 | 19.7 | **18.3** |
| VII | 19.6 | 19.4 | 20.9 | 19.2 | 18.5 | 20.9 | 19.9 | 21.0 | 19.2 | 19.7 | **19.8** |
| VIII | 18.0 | 19.3 | 19.9 | 19.5 | 17.9 | 19.6 | 17.3 | 20.2 | 19.3 | 19.8 | **19.1** |
| IX | 12.9 | 16.4 | 15.7 | 13.8 | 13.9 | 15.2 | 14.4 | 13.4 | 13.7 | 14.7 | **14.4** |
| X | 7.4 | 8.5 | 10.5 | 11.7 | 11.4 | 9.6 | 8.1 | 9.1 | 11.0 | 11.2 | **9.9** |
| XI | 7.8 | 1.8 | 8.5 | 5.8 | 8.0 | 4.6 | 6.3 | 4.9 | 6.0 | 9.5 | **6.3** |
| XII | -0.1 | 2.3 | 0.0 | 1.6 | 2.7 | 1.4 | -1.6 | 1.9 | 0.6 | 3.9 | **1.3** |
| **Average** | **9.2** | **9.2** | **10.0** | **9.9** | **10.9** | **9.9** | **10.1** | **9.9** | **10.0** | **10.7** | **10** |
