# Supplementary material for: Development of Neonectria punicea Pathogenic Symptoms in Juvenile Fraxinus excelsior Trees
Source: Front Plant Sci. 2020 Dec 23;11:592260. doi: 10.3389/fpls.2020.592260 (PMC7785714; doi:10.3389/fpls.2020.592260)
Supplement: Supplementary file 8 [file Table_2.DOCX]

**Supplementary Table S2.** Average precipitation in the studied locality, obtained from the TFA® NEXUS weather station for the 2010-2019 period.

| Months | 2010 | 2011 | 2012 | 2013 | 2014 | 2015 | 2016 | 2017 | 2018 | 2019 | **Average** |
| --- | --- | --- | --- | --- | --- | --- | --- | --- | --- | --- | --- |
| I | 226.5 | 86.5 | 80.3 | 183.5 | 91.4 | 145.6 | 84.5 | 45.6 | 93.6 | 102.8 | **114** |
| II | 118.4 | 49.6 | 91.8 | 234.8 | 90.5 | 123.1 | 196.8 | 92.4 | 250 | 42.2 | **129** |
| III | 142.2 | 52 | 17.8 | 119.1 | 151.1 | 107.6 | 151.2 | 122.4 | 104.8 | 71 | **103.9** |
| IV | 81.8 | 60.8 | 179.7 | 99.4 | 243.6 | 96.2 | 62.8 | 156.2 | 46.6 | 94 | **112.1** |
| V | 140.6 | 78.5 | 233.8 | 114.4 | 278.1 | 154.4 | 137.4 | 95.2 | 141.8 | 303.4 | **167.8** |
| VI | 304.9 | 84.1 | 48.6 | 56.1 | 101 | 96.4 | 149.8 | 56.4 | 184.8 | 115.6 | **119.8** |
| VII | 73.9 | 114.1 | 97.7 | 28.7 | 172 | 60.4 | 208 | 7.8 | 86.4 | 58.2 | **90.7** |
| VIII | 86.1 | 25.2 | 18.4 | 98.4 | 174.7 | 38.8 | 132.6 | 67.4 | 17.4 | 45.1 | **70.4** |
| IX | 320.8 | 44.5 | 150.8 | 69 | 344.2 | 146 | 147.4 | 204.6 | 48.4 | 142.1 | **161.8** |
| X | 132.5 | 100.3 | 129.7 | 70.1 | 125.6 | 203.2 | 129.4 | 116.4 | 15.4 | 39.7 | **106.2** |
| XI | 131.3 | 0.9 | 99 | 262.1 | 51.6 | 91.4 | 111.4 | 161.6 | 46.8 | 169.2 | **112.5** |
| XII | 106.8 | 135.3 | 268.4 | 22.3 | 120.8 | 12.8 | 10.6 | 156 | 79.6 | 142.8 | **105.5** |
| **Total** | **1865.8** | **831.8** | **1416** | **1357.9** | **1944.6** | **1275.9** | **1521.9** | **1282** | **1115.6** | **1326.1** | **1393.7** |
